# Manufacturing a TiO_2_-Based Semiconductor Film with Nanofluid Pool Boiling and Sintering Processes toward Solar-Cell Applications

**DOI:** 10.3390/nano12071165

**Published:** 2022-03-31

**Authors:** Saeid Vafaei, Ian Holmes, Benjamin Errion, Zigmey Thukka, Ryoki Narita, Takashi Sugiura, Kazuhiro Manseki

**Affiliations:** 1 Mechanical Engineering Department, Bradley University, 1501 West Bradley Avenue, Peoria, IL 61625, USA; 2 Graduate School of Natural Science and Technology, Gifu University, Yanagido 1-1, Gifu 501-1193, Japan

**Keywords:** nanofluid pool boiling, nanoparticles, crystallization, sintering, TiO_2_

## Abstract

For the first time, nanofluid boiling was applied as a process for the creation of a semiconductor TiO_2_ nanoparticle film that can be deposited onto a conductive substrate (F-doped SnO_2_ glass: FTO). A steel-base device designed for pool boiling was used to deposit a TiO_2_-based nanofluid consisting of nanoparticles with an average size of about 20 nm. The boiling of the nanofluid directly on the FTO glass substrate allowed for the deposition of the nanoparticles onto the FTO surface. In principle, the surface responsible for transferring heat to the fluid can be covered with these nanoparticles when the nanofluid boils. Using the as-deposited films, crystal growth of the TiO_2_ nanoparticle was controlled by varying the strategies of the post-sintering profile. The maximum temperatures, periods, and ramping rates for the obtained samples were systematically changed. Scanning electron microscopy (SEM) revealed that a densely packed TiO_2_-nanoparticle layer was obtained for the as-deposited substrate via pool boiling. For the maximum temperature at 550 °C, the TiO_2_ grain sizes became larger (~50 nm) and more round-shaped TiO_2_ nanostructures were identified. Notably, we have demonstrated for the first time how the sintering of TiO_2_ nanoparticles proceeds for the nanoporous TiO_2_ films using high-resolution transmission electron microscopy (TEM) measurements. We found that the TiO_2_ nanoparticles fused with each other and crystal growth occurred through neighboring 2–4 nanoparticles for the 550 °C sample, which was proved by the TEM analysis that continuous lattice fringes corresponding to the (101) anatase phase were clearly observed through the entire area of some nanoparticles aligned horizontally. In addition, the loss of the TiO_2_ nanofluid (precursor solution) was completely avoided in our TiO_2_ deposition. Unlike the commonly used spin-coating method, nanofluid pool boiling would provide an alternative cost-effective approach to manufacture semiconductor layers for various applications, such as solar cells.

## 1. Introduction

Successful deposition of nanoparticles on a substrate, such as conductive glasses, is a major issue in order to produce high-quality films applicable for versatile electronic applications. One of the most important issues includes the development of solar-energy conversion devices, as have been demonstrated in the fundamental studies on next-generation solar cells. In recent years, perovskite solar cells have received considerable attention due to their potential of being a low-cost alternative to conventional silicon-based solar cells, stemming mainly from the solution-processible characteristics and remarkably high light-to-electricity conversion efficiency [1]. In regards to such a photovoltaic application, TiO_2_-nanoparticle films have often been utilized as a scaffold for the light-absorber layers in perovskite solar cells, and those TiO_2_ porous films play an important role in the formation of a favorable material interface with the halide–perovskite compounds [1,2]. To ensure a high electron-transport efficiency in the TiO_2_ film, sintering at 450~500 °C is often used after TiO_2_ has been deposited on the conductive glass. As a result of this sintering, the joining of nanoparticles can be promoted. In regard to the TiO_2_ deposition, spin-coating methods have often been tested to fabricate perovskite solar cells. One of the major advantages for the spin-coat approach includes controllable and uniform thickness of the films by simply changing the coating strategies. The resultant films have a relatively planar surface. However, spin-coating has several major disadvantages including loss of nanoparticles, difficulty in deposition of particles with different sizes, and deposition of materials on top of each other in different layers.

**Figure 1:**
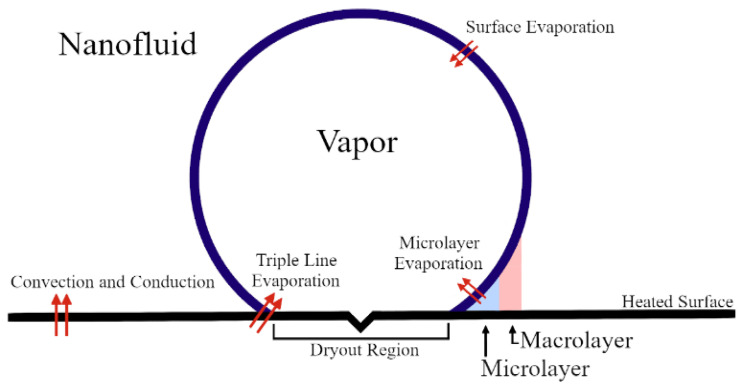
Bubble formation, characteristic heat transfer, and microlayer visualization.

We anticipated that nanofluid pool boiling would be an alternative process to deposit the nanoparticle layer onto the conductive glass substrate [3,4,5,6,7,8,9,10,11,12]. It should be noted that this method has an advantage of depositing the precursor solution without loss. A nanofluid is a base fluid with nanoparticles, where a surfactant often allows the dispersion of these nanoparticles to increase the liquid homogeneity. When this nanofluid boils, the surface responsible for transferring heat to the fluid will be covered with these nanoparticles. The microlayer, as visualized in Figure 1, sees a rapid spike in the nanoparticle concentration with the growing size of the vapor bubble. The increased concentration increases the probability of collision and agglomeration of nanoparticles. As the nanoparticles come together, they will fall out of solution and deposit onto the surface. The deposited nanoparticles produce a homogenous layer of nanoparticles laterally and in depth on the heated substrate. The deposition of nanoparticles on the heated substrate during the nanofluid boiling, and the homogeneity of the deposited nanoparticles, have been reported by many researchers, which can be seen in reference [13]. In this research, more than 50 samples were tested and it was observed that nanoparticles deposit uniformly, laterally, and in depth. An interesting additional benefit of the nanofluid-boiling process is that the heated surface will begin to sinter the thin deposited layer. Although this heat treatment is not enough to obtain the desired properties, it is enough to increase the stability of the deposited layer. By taking advantage of the stability of the film and the sintering properties of TiO_2_ nanoparticles, a post-sintering treatment after the fluid deposition has significant effects to create a robust film applicable for solar-cell applications. Such sintering properties for the TiO_2_ deposition that is assisted by nanofluid pool boiling, i.e., nanostructure characteristics of the sintered individual TiO_2_ particle, have not been clearly understood so far. In this paper, TiO_2_-based nanofluids were successfully deposited on a FTO substrate using the pool-boiling method with a steel-base device. In addition, sintering strategies, such as maximum temperatures and period of their temperatures, were systematically changed to assess the TiO_2_ crystal growth in the film.

Additionally, the focus of this paper lies in the application of the TiO_2_ layer for perovskite solar cells. Light absorption is the first step of light-to-electricity conversion in perovskite solar cells. Compounds such as methylammonium lead trihalide (CH_3_NH_3_PbX_3_) are the key light absorbers, where X denotes Br^−^ and/or I^−^. Perovskite materials show strong light absorption in a wide range of the visible light spectrum, leading to the generation of high photocurrents when they are combined with the porous layer of TiO_2_ particles (acting as an electron-extracting layer) and a hole-conducting layer. The preliminary evaluation of the obtained TiO_2_ layer in perovskite solar cells is provided.

## 2. Materials and Methods

### 2.1. Chemicals

Ethanol (Wako, Chicago, IL, USA or Kanto Kagaku, Tokyo, Japan, 99.5%), conc. HNO_3_ solution (Wako, Chicago, IL, USA) and titanium tetraisopropoxide (Kanto Kagaku, Tokyo, Japan) were used as purchased. A commercial nanocrystalline TiO_2_ paste (Sigma Aldrich, St. Louis, MO, USA, Greatcell Solar^®^) was also used. The TiO_2_ paste has a TiO_2_ concentration of ~17%, viscosity of ~170,000 mPa·s, mean pore diameter of 18 nm, and porosity of 60%. SnO_2_ nanoparticles were synthesized using the following low-temperature sol-gel method, previously reported in our group [14]. Briefly, 500 mg of SnCl_2_ was mixed into 100 mL of ultrapure water (UP-water) using sonication. A total of 100 mg of Na_2_CO_3_ dissolved in a separate beaker filled with 100 mL of water was then added and completely mixed. This mixture was maintained at 40 °C for 48 h to form tiny nanocrystals with approximate sizes of 5 nm. After centrifugation of the reaction mixture, the obtained solid gel was mixed with 5–10 mL of water and sonicated. This sample was then treated with vacuum freeze-drying apparatus at ~200 mTorr. SnO_2_ nanoparticles were produced in a powdered form. 

### 2.2. Preparation of Nanofluids

The composition of the nanofluid solution that was used in the nanofluid-boiling deposition experiment was comprised of 0.05 g of SnO_2_ and 2 g of premixed TiO_2_ paste (comprised of 75 wt% ethanol and 25 wt% TiO_2_) combined with 31.33 g of ethanol. This mixture has a final composition of 0.15 wt% SnO_2_, 1.5 wt% TiO_2_, and 98.35 wt% ethanol. To ensure the proper mixing of the nanofluid components, the solution was placed into a 3-L ultrasonic cleaner to undergo a 10 min sonication cycle. In order to ensure a consistent nanoparticle concentration and deposition during the boiling process, a high volume of nanofluid (~50 mL) was used. The use of high volumes of nanofluid reduces the change of nanoparticle concentration in the nanofluid due to evaporation in the boiling process. Nanoscale SnO_2_ particles were mixed with the TiO_2_ precursor solutions in order to ensure that an electron-transport path applicable for photovoltaic applications was created.

### 2.3. Preparation of Nanofluid-Boiling Device

**Figure 2:**
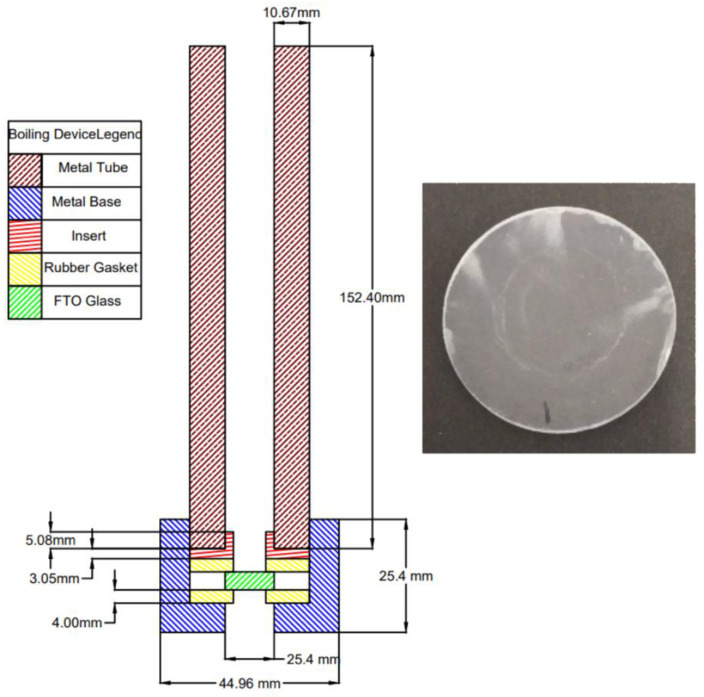
Section view of the nanoparticle-boiling deposition device and a typical image of FTO glass substrate after TiO_2_ deposition.

A stainless-steel tube with a base was used to boil the nanofluid directly over the surface of an FTO glass substrate (Sigma-Aldrich, St. Louis, MO, USA, resistivity: ~7 Ω/sq.), which was cut into a circle with a diameter of 3.4 cm. The boiling of the nanofluid directly on top of the FTO glass substrate allowed for the deposition of the nanoparticles onto the surface of the substrate. The nanofluid-boiling device is depicted in Figure 2 and is composed of five pieces: a steel tube, steel base, insert, rubber gaskets, and the FTO glass substrate.

The base has an axial hole that was designed to allow an FTO substrate sample to seal the bottom of the tube. Rubber gaskets ensure that no fluid can leak out of the bottom or around the substrate and allow the entire apparatus to remain functional, even with thermal expansion. This hole, completed by the substrate, allows for direct heating of the substrate and provides a way for operators to easily check on the deposition process. The insert provides a flat surface in which the rubber gaskets and FTO glass substrate can contact to ensure proper sealing around the substrate. The tube and base are threaded such that the tube can be screwed into the base, providing a force for the insert and gaskets to seal against. This coupling allows the tube to become a reservoir for the nanofluid to be boiled. The threads of the pipe were also coated in Teflon tape to ensure that the nanofluid cannot leak out of the threaded connection between the pipe and the base. A Bunsen burner was used to provide heat to the apparatus and promote the boiling process.

### 2.4. Sintering Process

Altering the parameters of the sintering process can have drastic effects on the structures. To investigate this, the parameters of temperature ramping rate, hold temperature, and hold time were changed. It is important to understand the definition of each parameter to begin to correlate its effects on the resulting product. Although the parameter of temperature ramping rate is the first in the physical process, its end point, hold temperature, must be decided first. Hold temperature is the peak temperature that the sample will endure during the process. Hold time is the time that this temperature must be sustained. Finally, the ramping rate is described as the change in temperature over a period of time to meet the hold temperature. The sintering was carried out in air. The hold temperatures used were 250, 300, and 550 °C. The temperature ramping rate was 1 or 15 °C/min. Hold times at these temperatures were 1 or 6 h. For comparison purposes, one sample was left entirely unsintered. For solar cells, the substrate was sintered at 500 °C as reported previously [15]. Three substrates for different solar cells (Devices 2–4 as mentioned in the following section) were prepared.

### 2.5. Evaluation of TiO_2_ Films

Nanostructures of TiO_2_ films after nanofluid pool boiling were characterized by scanning electron microscopy (SEM; S-4800) (HITACHI, Tokyo, Japan) and transmission electron microscopy (TEM; JEM-2100) (JEOL, Tokyo, Japan). For the SEM images, the longest axis of particles was measured to analyze the size distribution in the films. Both unsintered and sintered substrates were characterized using *x*-ray diffraction (XRD; Rigaku RINT Ultima/PC with monochromated Cu Kα radiation, Tokyo, Japan). Except for the perovskite layer, the solar cell was assembled using the method that has been previously reported in our group, where TiO_2_ substrates were sintered in air at 500 °C [15]. In regards to the TiO_2_-based electron-transport layer (ETL) in perovskite solar cells, a TiO_2_/FTO substrate was prepared, using the spin-coating process and sintering at 500 °C for comparison (Device 1). For the nanofluid-boiling samples, Device 2 was fabricated using the TiO_2_/SnO_2_ substrate treated with 500 °C sintering. Devices 3 and 4 had pure TiO_2_ or TiO_2_/SnO_2_ substrate, for which the surface was further coated using the following precursor solution to form a compact TiO_2_ layer at the top. 13 mL of ethanol was mixed with 0.34 mL of UP water and 2 drops of a concentrated HNO_3_ solution were then added and stirred at room temperature. A total of 1mL of Titanium tetraisopropoxide (TTIP) was further added to the mixture. Lastly, 2 drops of HNO_3_ were added to prepare the precursor solution. To deposit the compact TiO_2_ layer, 0.25 mL of obtained precursor solution was dropped on the surface of the TiO_2_/SnO_2_ substrate, followed by spin-coating at 1500 rpm for 30 s and 1000 rpm for 60 s. The deposited substrate was dried on a hotplate at 100 °C for 30 min and the substrate was treated in air at 100 °C for 50 min and 500 °C for 30 min.

For the light-absorber layer, the mixed-cation perovskite, Cs_0.05_MA_0.1_FA_0.85_PbI_2.9_Br_0.1_·0.05 PbI_2_ (MA: methylammonium, FA: formamidinium) was used for all 4 devices (Devices 1–4) using the reported method (control experiment with FABr addition) [16]. Current-voltage curves were obtained under AM 1.5 simulated sunlight (100 mW/cm^2^) using a solar simulator (Yamashita Denso, YSS-80A, Tokyo, Japan) and a potentiostat (Hokuto Denko, Tokyo, Japan, HSV-110). The active area of the device was regulated to be 0.3 cm × 0.3 cm.

## 3. Results and Discussions

In regards to the nanofluid pool boiling, Modi et al. [17] explained that nanoparticle deposition happens as a result of evaporation in the microlayer beneath the bubble, where most of the heat and mass transfer occurs. Li et al. [18] also reported that the microlayer accumulates nanoparticles and deposits them when the microlayer fully evaporates. In their experiments, microlayer evaporation was the key process to bubble growth and that theoretically, the deposition process would continue for as long as the nanofluid is boiling. Kim et al. [19] also hypothesized that nanoparticles are deposited from the microlayer formed underneath the vapor bubble during nanofluid boiling. In order to test the hypothesis, an experiment was conducted using a copper heater which was submerged in Al_2_O_3_ nanofluid. Power was increased to the heater so that a single nucleation site was formed on the surface of the heater. The power was then held constant and the single active nucleation site underwent several boiling cycles. After 2 min of boiling, the power was shut off and the surface of the heater was inspected where the single nucleation site was. It was observed that a single, circular nanoparticle film was formed at the single active nucleation site and no other nanoparticle deposition was observed on the heater surface. With this study it was clearly demonstrated that nanofluid boiling and evaporation of the microlayer is the responsible mechanism for nanoparticle deposition.

**Figure 3:**
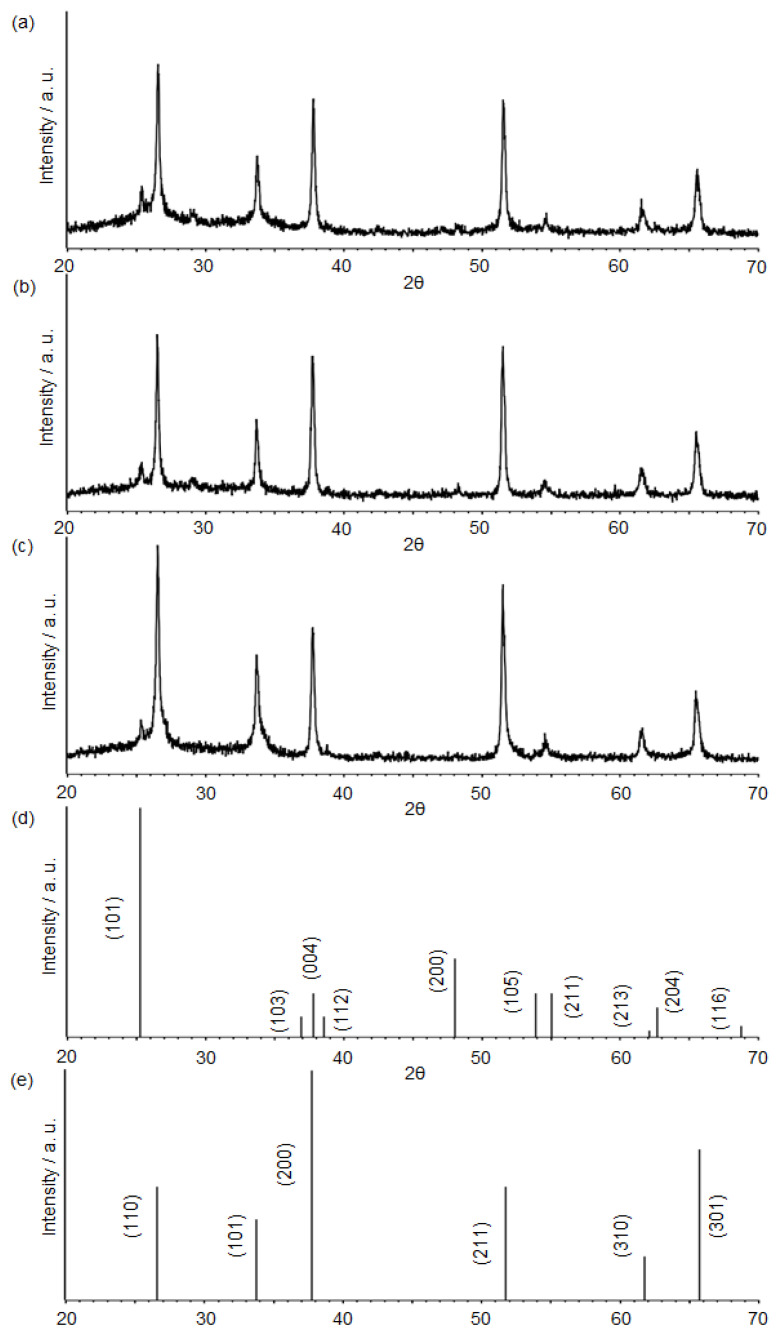
(**a**) XRD pattern of the as-deposited TiO_2_ film on FTO substrate, formed by nanofluid pool boiling. (**b**) XRD pattern of the heat-treated sample at 250 °C. The ramping rate and period of the temperature were 1 °C/min, 1 h, respectively. (**c**) XRD pattern of the sintered sample at 550 °C. The ramping rate and period of the temperature were 15 °C/min, 1 h, respectively. (**d**,**e**) present the database of SnO_2_ (JCPDS: 00-046-1088) and TiO_2_ (JCPDS: 00-021-1272), respectively.

For the as-deposited sample after pool boiling, the *x*-ray diffraction (XRD) pattern in Figure 3a indicated the anatase phase TiO_2_ which originated from the TiO_2_ nanofluid, showing a main peak at 25.3°. This was assigned to the (101) crystal plane of anatase TiO_2_. The anatase TiO_2_ phase corresponding to the (101) plane was also identified for heat-treated samples. Examples of those XRD pattern are depicted in Figure 3b,c (Figure 3b: maximum temperature: 250 °C, ramping rate: 1 °C/min, period of maximum temperature: 1 h, Figure 3c: maximum temperature: 550 °C, ramping rate: 15 °C/min, period of maximum temperature: 1 h). Since F-doped SnO_2_ (FTO) substrate glass was used for the pool deposition, larger SnO_2_ peaks in the XRD pattern were most likely from the FTO surface. All the observed TiO_2_ and SnO_2_ peaks in Figure 3a–c were consistent with their database (Figure 3d,e). The crystallite size of the TiO_2_ particles of Figure 3c sample was estimated from the Scherrer Equation (1):

(1) $$D=\frac{K\lambda }{\beta \cos\theta }$$

D, K, λ, and θ indicate the crystallite size of particles, Scherrer constant (=0.90), *x*-ray wavelength (=1.54 Å), and Bragg angle, respectively. The estimated crystallite size was found to be 42 nm, being consistent with the observed average size of 43 nm from the corresponding SEM image, as discussed below.

**Figure 4:**
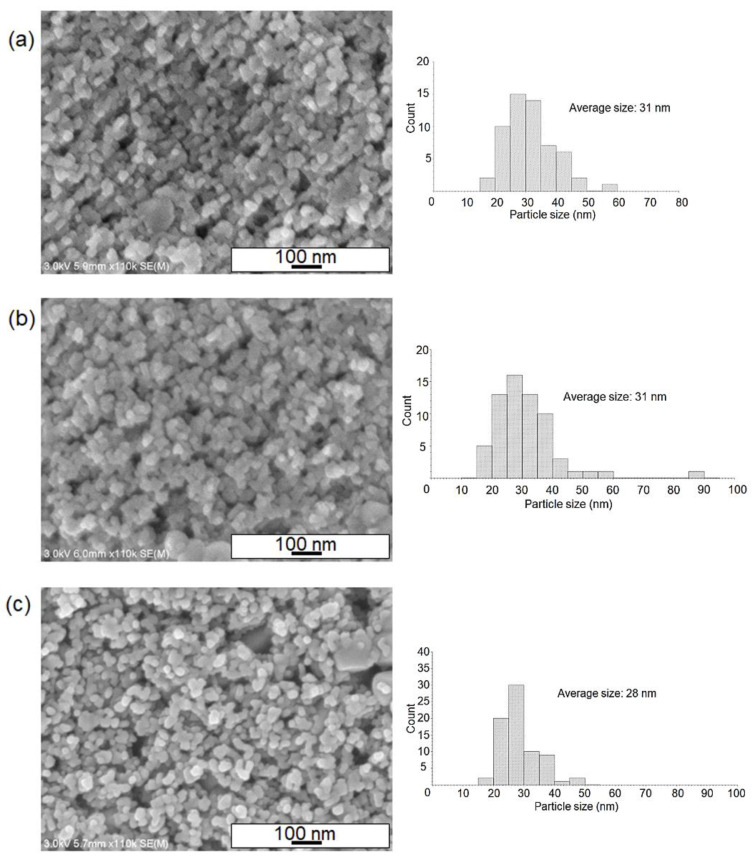
SEM images of the surface of TiO_2_ films after nanofluid pool boiling, where the post sintering strategies have been changed. Sintering strategies (maximum temperature, ramping rate, and the period of maximum temperature): (**a**) without sintering, (**b**) 250 °C, 1 °C/min, 1 h, (**c**) 300 °C, 1 °C/min, 6 h. Particle size distributions of the nanoparticles were also presented.

**Figure 5:**
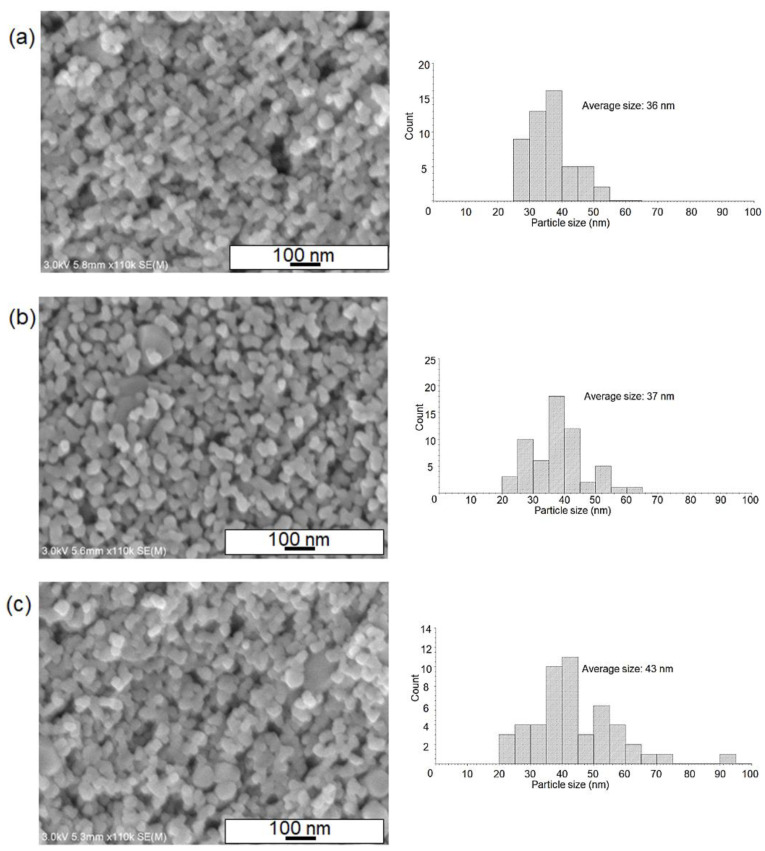
SEM images of the surface of TiO_2_ films after nanofluid pool boiling, where the post-sintering strategies have been changed. Sintering strategies (maximum temperature, ramping rate, and the period of maximum temperature): (**a**) 550 °C, 1 °C/min, 1 h, (**b**) 550 °C, 1 °C/min, 6 h, (**c**) 550 °C, 15 °C/min, 1 h. Particle size distributions of the nanoparticles were also presented.

To understand the nanostructures of both the as-deposited and sintered films, we investigated the SEM images of samples with changes in the temperature profiles as presented in the experimental part. As shown in Figure 4a, the SEM image of the surface of the obtained film (without sintering) indicated the formation of densely-packed TiO_2_ nanoparticles layer after the nanofluid deposition. It is most likely that the particles of around 20 nm in size were the ones from the commercial TiO_2_ precursor paste. Figure 4b,c revealed that the post-sintering process at relatively lower temperatures produced a porous TiO_2_ surface with increased pore sizes when compared to the unsintered substrate (Figure 4a). For these conditions at the temperature of 250~300 °C, it appears that the neighboring nanoparticles started to join with each other, and at the same time, TiO_2_ paste compositions such as organic binders decomposed and evaporated in the heating process. This most likely led to the enhancement of pore size in the TiO_2_ layer. For the maximum temperature at 550 °C, the TiO_2_ grain sizes became larger (~50 nm) and more round-shaped nanostructures were identified (Figure 5a–c). Regarding SEM images of both un-sintered and sintered samples after nanofluid pool boiling, we analyzed a particle size distribution for all data in order to understand sintering effects. From the histograms as shown in Figure 4 and Figure 5, the average particle sizes for 550 °C treatments (Figure 5a–c) were found to be larger than those of low-temperature-processed samples. These results mean that the crystal growth of TiO_2_ nanoparticles occurred in the temperature ranges of around 300~550 °C.

**Figure 6:**
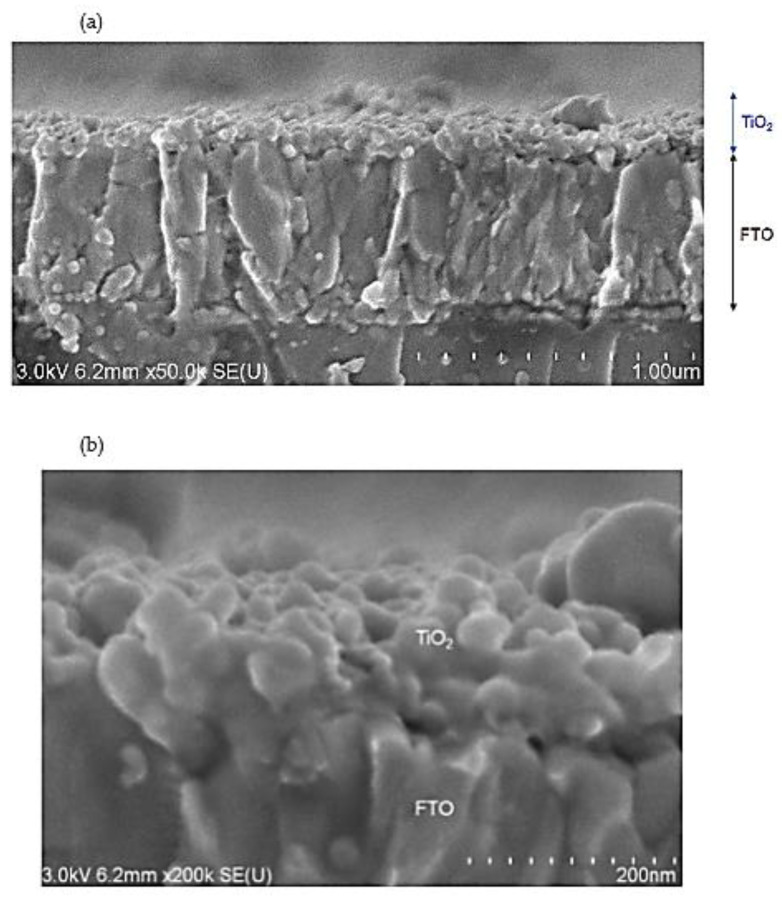
(**a**,**b**) Cross-sectional SEM images of an as-deposited TiO_2_ film after nanofluid pool boiling. (**b**) High-magnification image of the same sample.

To gain insight into the lateral structure of the deposited film, we measured a cross-section SEM image of the TiO_2_/FTO substrate (Figure 4a sample), as shown in Figure 6. The measurements indicated the formation of an uneven surface after the pool boiling, with the approximate thickness ranging from 50–200 nm (Figure 6a). A higher-magnification image of Figure 6b also supported the formation of the sintered TiO_2_ nanoparticles of ~40 nm in size, as discussed in the SEM data analysis.

**Figure 7:**
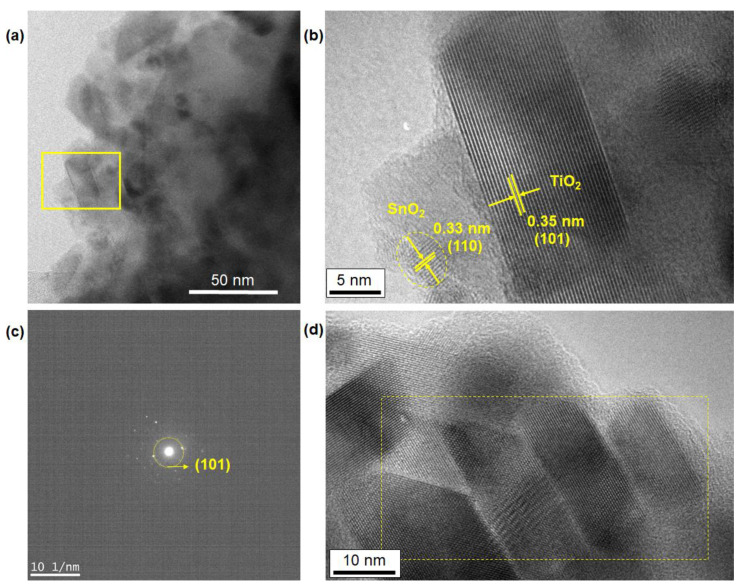
(**a**,**b**) TEM images of the as-deposited film after nanofluid pool boiling. The yellow part shown in (**a**) corresponds to the position of the high-resolution TEM (HR-TEM) image of (**b**). (**c**) The selected-area diffraction pattern indicating the diffraction spots of the 101 plane of the TiO_2_ particles that correspond to the image (**b**). (**d**) HR-TEM image of the sintered sample at 550 °C (ramping rate: 15 °C/min, period of maximum temperature: 1 h). The area shown in a dotted yellow line indicates the joining of some TiO_2_ nanoparticles as a result of the sintering process. The spacing of each lattice fringe, 0.35 nm, of the TiO_2_ anatase phase is omitted for clarity.

Figure 7 shows TEM images of the as-deposited film after nanofluid pool boiling. The area of the yellow line highlighted in Figure 7a presents the same position of the high-resolution TEM image of Figure 7b. The anatase crystal phase of TiO_2_ nanoparticles with the dimensions of around 20 nm was identified from the analysis of the lattice fringes in image Figure 7b. In addition, a SnO_2_ nanoparticle with a size of 5 nm was also observed in the same image Figure 7b. The selected-area diffraction pattern (SAD) in Figure 7c indicated the (101) plane of the TiO_2_ particles. The yellow part in Figure 7d, which is the sintered sample at 550 °C, indicated the joining of some TiO_2_ nanoparticles as a result of the sintering process. Notably, continuous lattice fringes corresponding to the (101) anatase phase were clearly observed through the entire area of some nanoparticles aligned horizontally. To our knowledge, this is the first finding that reveals the structure of the interparticles of the sintered nanoporous TiO_2_ films at a single-nanometer scale.

Most importantly, the loss of nanoparticles was completely avoided for producing a porous TiO_2_ film, whose uniform film quality at the scale of tens of nanometers is similar to the previously reported films that were prepared using conventional wet processes, such as spin-coating [1,20]. Unlike the spin-coating method, in nanofluid-boiling nanoparticle deposition, the nanoparticles remain in the container and never spread around, which is important from cost and safety points of view.

**Figure 8:**
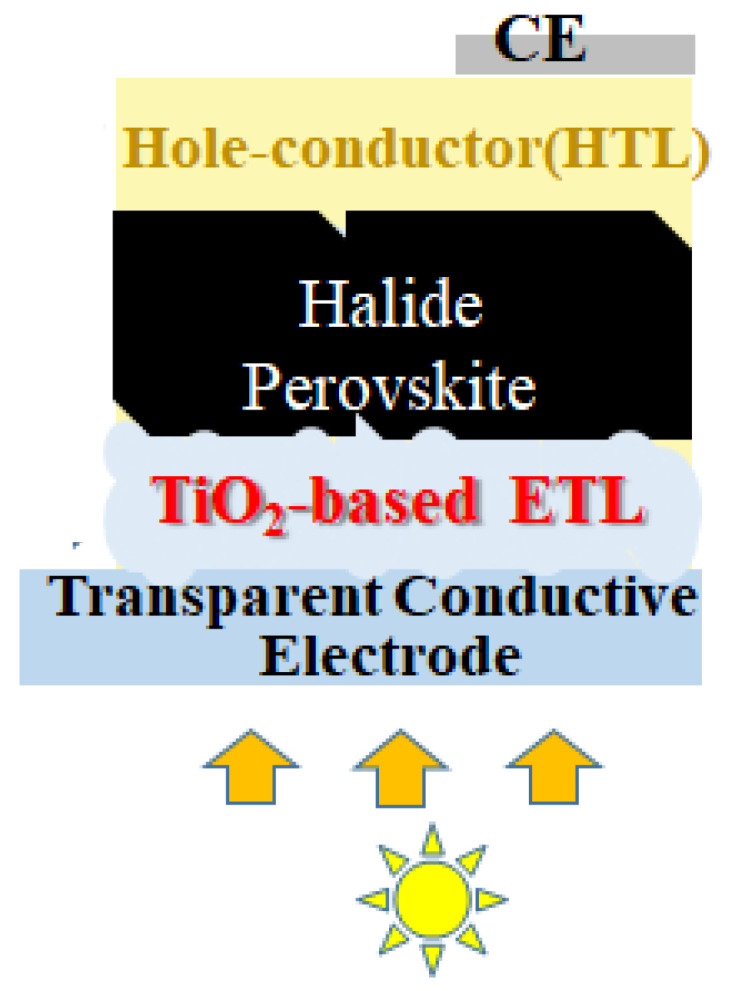
A device structure of perovskite solar cells using a TiO_2_-based scaffold that was deposited on a transparent conductive electrode (FTO). ETL, HTL, and CE denote electron-transport layer, hole-transport layer, and counter electrode (Au), respectively.

The sintered TiO_2_ film was examined as a scaffold to deposit a lead-halide perovskite compound to test the performance of the perovskite solar cell, where the perovskite absorbs incident light and the adjacent TiO_2_ works as an electron-extracting layer in the device (Figure 8). I–V parameters of short-circuit current density (J_sc_), open-circuit voltage (V_oc_), fill factor (FF), and light-to-electricity conversion efficiency (η) were obtained for the device. The η value was obtained using the following Equation (2).

(2) $$\eta =\left(\mathrm{iph}\times V_{\mathrm{oc}}\times \;\mathrm{FF}\right)/I$$

The iph and I correspond to the integral current density and intensity of incident light (I = 1000 W/m^2^), respectively. The preliminary test of the TiO_2_/SnO_2_ substrate deposited using pool boiling in Table 1 showed a relatively low efficiency of 6.63% (Device 2), when compared to that of the control experiment using the conventional spin-coat method (Device 1). The decreased efficiency is probably due to the uneven surface of the TiO_2_/SnO_2_ layer as presented in Figure 6, leading to the imperfect connection between the ETL and crystallized perovskite after its perovskite spin-coat process. In fact, an additional compact TiO_2_ layer (one-time coat-process of TiO_2_) formed on top of both pure TiO_2_ (Device 3) and TiO_2_/SnO_2_ (Device 4) ETLs had a tendency to improve the light-to-electricity conversion efficiency. Optimization of the lateral structure at a nanoscale using nanofluid pool boiling will provide useful criteria for ETL to further improve the solar cells’ performance without the loss of costly materials such as nanocrystalline-TiO_2_ pastes.

## 4. Conclusions

We have demonstrated for the first time that sequential pool boiling and sintering processes are alternative processes to produce uniform porous TiO_2_ layers. Using high-resolution TEM measurements we have, for the first time, shown how the sintering of TiO_2_ nanoparticles proceeds for nanoporous TiO_2_-based films. Most importantly, the loss of the TiO_2_ nanofluid can be completely avoided in our TiO_2_ deposition. Nanofluid pool boiling would be a beneficial, cost-effective approach in manufacturing semiconductor layers for photovoltaic applications, including not only perovskite solar cells but also dye-sensitized solar cells.

## Figures and Tables

**Table 1:** Photovoltaic performance of the perovskite solar cells under a simulated solar light. The best-performing data from the reverse scan were summarized for each device.

	J_sc_ (mA/cm^2^)	V_oc_ (mV)	FF	η (%)
Device1	25.0	1060	0.670	17.8
Device2	16.1	890	0.462	6.63
Device3	21.5	890	0.428	8.19
Device4	19.5	930	0.488	8.86

## Data Availability

The data presented in this study are available on request from the corresponding author.
